# Spinal Subdural Abscess in the Emergency Department: A Rare Diagnosis in an Immunocompetent Host

**DOI:** 10.1016/j.acepjo.2026.100499

**Published:** 2026-09-18

**Authors:** Hashem Nassereddine, Jordan Richardson, Michael F. Sobin

**Affiliations:** 1 Department of Emergency Medicine, Corewell Health William Beaumont University Hospital, Royal Oak, Michigan, USA; 2 Department of Emergency Medicine, Medical College of Wisconsin; Milwaukee, Wisconsin, USA

**Keywords:** spinal subdural abscess, spinal epidural abscess, cauda equina syndrome, spinal infection, immunocompetent host, back pain, emergency department

## Abstract

Spinal subdural abscess is an exceptionally rare and potentially life-threatening spinal infection characterized by the accumulation of purulent material between the dura and arachnoid mater. In contrast to the more common spinal epidural abscess, spinal subdural abscesses have been reported in fewer than 80 cases, often in pediatric, postoperative, or immunocompromised patients. This case represents the fourth documented instance of a spinal subdural abscess in an immunocompetent adult without identifiable risk factors or a known source of infection and the first reported in emergency medicine literature. A 60-year-old immunocompetent woman presented to the emergency department with atraumatic low back pain initially attributed to lumbar radiculopathy. She returned six days later with worsening bilateral radicular pain, urinary incontinence, and systemic symptoms. Magnetic resonance imaging (MRI) revealed a large subdural fluid collection from T2 to L5, concerning for spinal subdural abscess. Emergent surgical decompression revealed frank purulence within the subdural space. Cultures grew *Streptococcus intermedius*, a rare pathogen in spinal infections. The patient completed an 8-week course of intravenous cefazolin and recovered fully with resolution of neurologic deficits. Spinal subdural abscesses can progress rapidly, causing widespread neurologic impairment due to the continuity of the subdural space. This case underscores the importance of maintaining a high index of suspicion for spinal infections in patients presenting with back pain and neurological deficits, even in immunocompetent patients. Early MRI and surgical consultation are essential to prevent irreversible neurological damage and optimize patient outcomes.

## 1 Introduction

Spinal subdural abscess, a purulent collection located between the dura and arachnoid mater, is an exceedingly rare entity, with fewer than 80 cases described in the literature.^1^, ^2^, ^3^, ^4^ These spinal infections are uncommon but important causes of cauda equina syndrome.^5^ Although most cases of cauda equina syndrome are attributed to compressive etiologies, infectious causes such as spinal epidural abscesses remain rare, with an estimated incidence of 0.2 to 2 cases per 10,000 hospital admissions annually.^5^^,^^6^ Although spinal epidural abscesses are the most recognized spinal space infection, the epidural space is not the only potential site for infection. Due to the unique anatomy of the subdural space, clinical presentations may differ from those of more common spinal infections. Most reported cases of spinal subdural abscesses have occurred in pediatric, postoperative, or immunocompromised patients.^1^, ^2^, ^3^, ^4^ We present the fourth reported case of a patient with spinal subdural abscess in an immunocompetent adult without identifiable risk factors or an apparent source of infection and the first in emergency medicine literature.^1^

## 2 Case Report

A 60-year-old woman with a past medical history of hypertension, obstructive sleep apnea, and migraines presented to the emergency department (ED) with right-sided atraumatic low back pain. The patient reported a 1-week history of pain radiating down her right leg, which was exacerbated by movement and relieved by rest. She denied saddle anesthesia, bowel or bladder dysfunction, paresthesia, extremity weakness, or fever. Her vital signs included blood pressure of 138/75 mmHg, heart rate of 74 bmp, temperature of 37 °C, respiratory rate of 18 rpm, and a room air SpO_2_ of 93%. On physical exam, she had 5/5 strength and normal sensation in her lower extremities without saddle anesthesia. She had mild tenderness to palpation of the right lumbar paraspinal muscles but no midline spinal tenderness. X-ray of her lumbar spine did not reveal any acute fracture or subluxation. Laboratory tests, including a complete blood count and basic metabolic panel, were obtained and were unremarkable. After administration of acetaminophen 1000 mg, ibuprofen 600 mg, and methocarbamol 500 mg, the patient reported improvement in her pain. The patient was discharged home on 40 mg oral prednisone with instructions to follow up with her primary care physician for presumed lumbar radiculopathy.

The patient returned to the ED 6 days later for worsening low back pain. During that time, she had developed increasing bilateral radicular leg pain and weakness, along with constipation and urinary incontinence. Vital signs were temperature 38.9 °C, heart rate 98 bmp, blood pressure 139/71 mmHg, and room air SpO2 95%. Physical examination demonstrated severe midline lumbar tenderness. A comprehensive neurological examination revealed 4/5 hip and plantar flexion strength in the left lower extremity and 4/5 strength in knee extension and dorsiflexion bilaterally. Patellar reflexes were hyperreflexic bilaterally; Babinski reflexes were downgoing and four beats of ankle clonus were appreciated bilaterally. Rectal tone was normal, but peri-anal sensation was decreased. A postvoid residual (PVR) urine volume was 650 mL, and a Foley catheter was placed. The remainder of her physical exam was unremarkable.

Blood cultures were obtained, and empiric intravenous (IV) vancomycin 2000 mg and piperacillin/tazobactam 4.5 g were started. Emergent magnetic resonance imaging (MRI) of the entire spine and orthopedic spine and neurosurgery team consultations were obtained due to suspicion for cauda equina syndrome.

Laboratory results were remarkable for a leukocytosis 14,200 10^9^/L (normal 3.3 to 10.7 × 10^9^/L) with an absolute neutrophil count of 11,970 10^9^/L (normal 1.60 to 7.20 × 10^9^/L), erythrocyte sedimentation rate 74 mm/h (normal 0 to 18 mm/h), and C-reactive protein of 390 mg/L (normal <8.0 mg/L).

MRI of the cervical, thoracic, and lumbosacral spine demonstrated enhancing epidural and posterior paraspinal fluid collections arising from the right L5-S1 facet with significant surrounding inflammatory enhancement, concerning for an infected synovial cyst versus epidural abscess (Fig 1). Additionally, an extensive rim-enhancing subdural collection was seen extending from the T2-T3 to L3-L4 level, causing mild mass effect and mild canal stenosis upon the thoracic cord, conus, and cauda equina nerve roots (Fig 2). Lastly, radiologic findings were suggestive of arachnoiditis, with diffuse enhancement of the cauda equina nerve roots.

**Figure 1:**
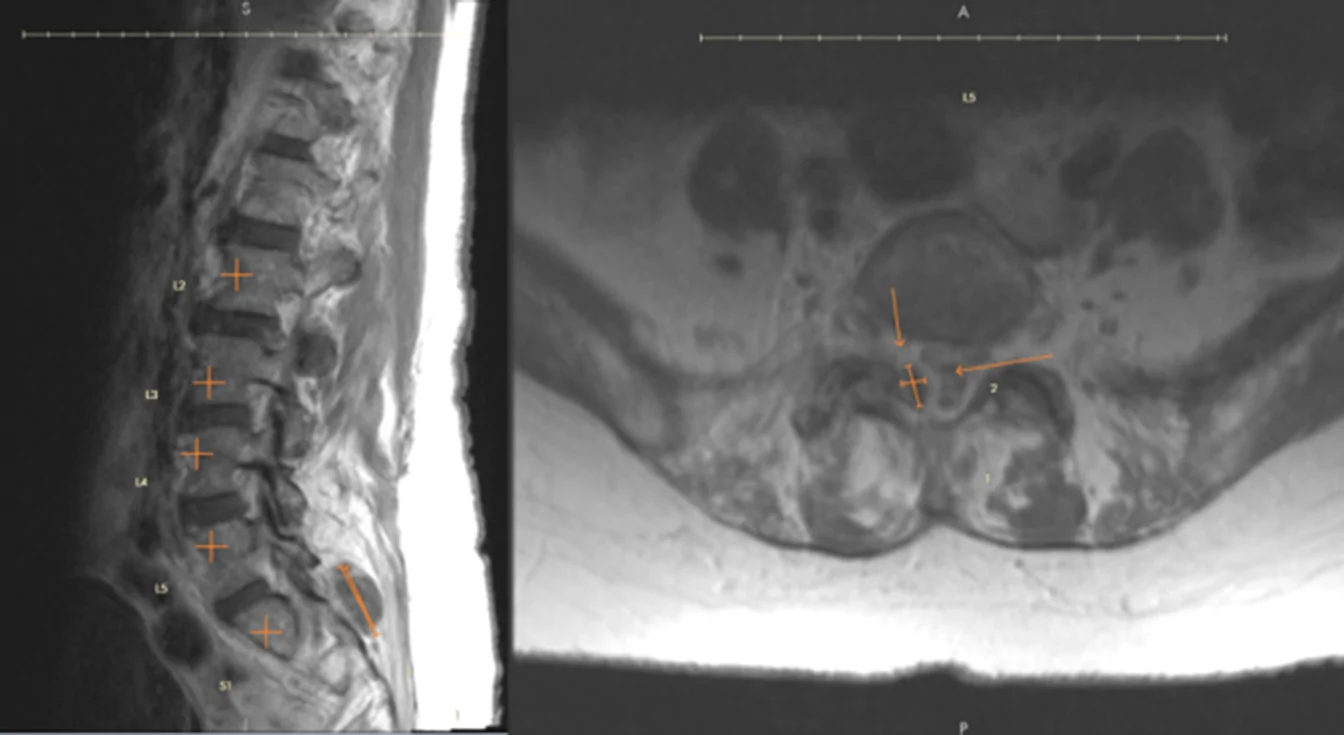
Sagittal and axial magnetic resonance images of the lumbosacral spine demonstrating enhancing epidural and posterior paraspinal fluid collections arising from the right L5–S1 facet with surrounding inflammatory changes, suggestive of an infected synovial cyst versus epidural abscess.

**Figure 2:**
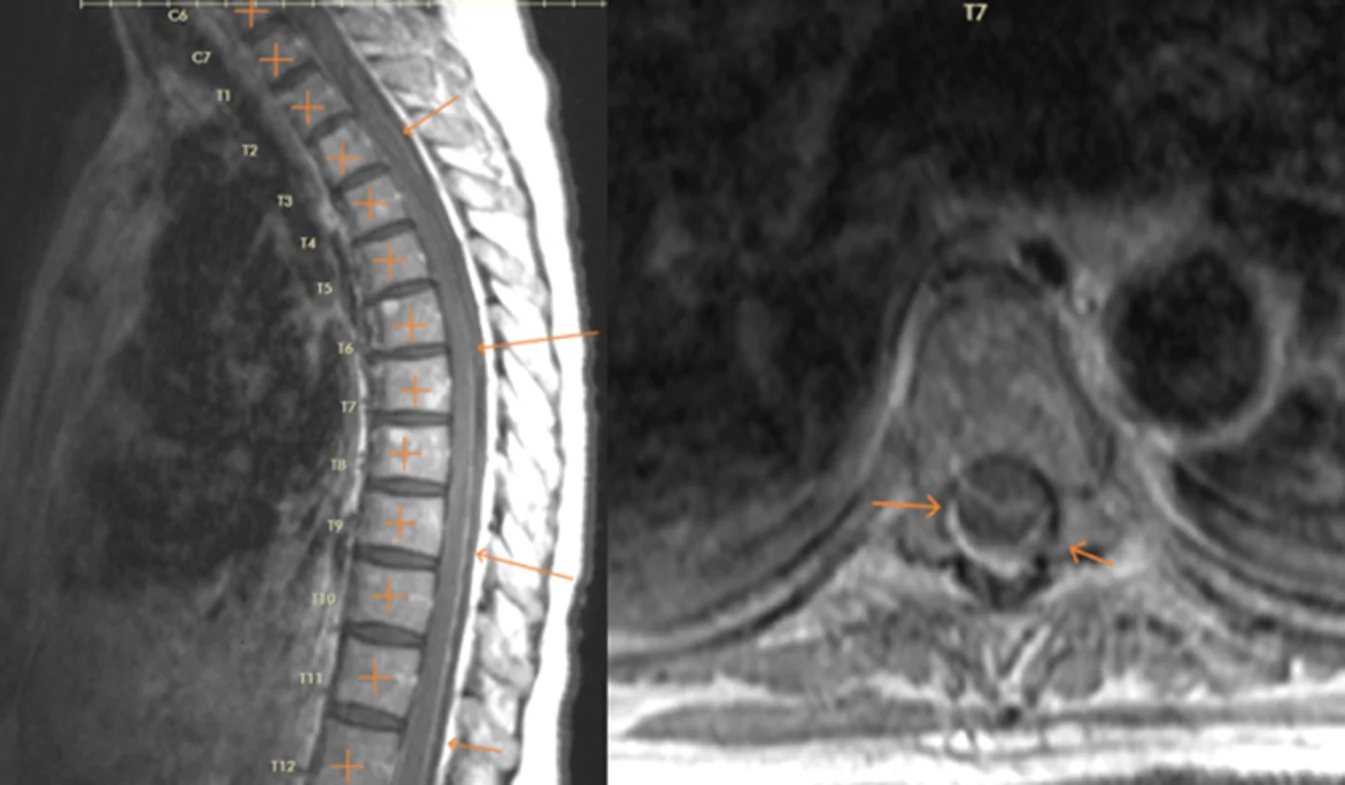
Sagittal and axial magnetic resonance images of the thoracic spine demonstrating an extensive rim-enhancing subdural collection which extends from T2–T3 to L3–L4, with mild mass effect and canal stenosis involving the thoracic cord, conus medullaris, and cauda equina nerve roots.

In discussion with both the orthopedic surgery and neurosurgery teams, all agreed that the patient would undergo emergent laminectomy by orthopedic surgery. The patient was taken to the operating room where a bilateral laminectomy from T12 to L5 was performed. This showed no signs of an epidural abscess; however, a durotomy at L1–L2 released purulent material, confirming a subdural abscess, while cerebrospinal fluid appeared clear. Additional durotomies at T6–T8 and L4–L5 drained further purulent fluid. The patient was hospitalized for 17 days under multidisciplinary care. Operative cultures grew *Streptococcus intermedius*, while blood cultures remained negative. Her antibiotic regimen was narrowed to IV cefazolin 2 g every 8 hours by the infectious disease team. A transthoracic echocardiogram did not demonstrate any evidence of endocarditis. Additionally, neither immunosuppressive conditions, procedures predating her presentation, nor a clear infectious source was identified. A postoperative incisional seroma was detected on repeat MRI following ongoing back pain and serous drainage at the incision site. The patient underwent wound exploration in the operating room; a small amount of serous fluid was evacuated, the cavity was irrigated, and topical vancomycin powder was administered. The patient had excellent postoperative neurological recovery. She regained normal bowel and bladder function, had full resolution of her motor and sensory deficits, and was ambulatory without assistance. She was discharged on hospital day 17 with infectious disease follow-up.

## 3 Discussion

A spinal subdural abscess is an exceedingly rare infection characterized by the accumulation of purulent material in the subdural space. This condition is distinct from the more commonly encountered spinal epidural abscess, which occurs in the epidural space between the dura mater and the vertebral column. Additionally, while spinal epidural abscesses are rare, affecting approximately 0.2 to 2 cases per 10,000 hospital admissions, spinal subdural abscesses are even more uncommon, with fewer than 80 cases described in the literature.^5^^,^^6^ Both conditions carry significant morbidity and mortality (13% to 16%), particularly when diagnosis and treatment are delayed conveying an odds ratio of permanent weakness of 5.7.^7^^,^^8^ In one case series of 119 spinal epidural abscess patients, over half were initially misdiagnosed, with a median delay of 12 days to appropriate diagnosis.^9^ Delayed identification may lead to irreversible neurologic deficits, sepsis, or death, underscoring the need for early recognition in the emergency setting.^10^

Infections of the spinal canal typically occur in the context of immunocompromised states or postinvasive spinal procedures. These risk factors often serve as clinical red flags that guide emergency physicians toward consideration of a spinal infection in the appropriate clinical context. However, the diagnosis becomes significantly more challenging in immunocompetent individuals without predisposing risk factors. Spinal subdural abscesses in this population are particularly elusive, as they lack the typical epidemiological cues that would prompt early consideration of this diagnosis.^3^ To our knowledge, this case represents only the fourth reported instance of a spinal subdural abscess occurring in an immunocompetent patient.^11^

The pathophysiology of spinal subdural abscesses is not well understood; they typically manifest with nonspecific systemic symptoms alongside focal neurological deficits. Patients experience severe, constant, localized back pain that is unresponsive to standard analgesics, with fever and other constitutional symptoms raising concern for an infectious process. Neurological deficits, such as radicular pain, motor weakness, sensory disturbances, and bowel or bladder dysfunction, vary according to the level and extent of involvement. In immunocompetent individuals without classical risk factors, symptoms may develop insidiously, potentially delaying diagnosis.^4^^,^^5^^,^^10^

Spinal epidural abscesses occur between the dura mater and vertebral canal and often present with localized pain until significant mass effect develops. In contrast, spinal subdural abscesses lie in the narrow space between the dura and arachnoid mater. They most commonly occur in the lumbar spine, although thoracic cases have been reported.^1^ Additionally, spinal epidural abscess is distinct from meningitis, given that the infection does not invade the meninges. This subdural compartment extends uninterrupted from the foramen magnum down to the sacrum, aside from minute openings for vessels and nerves. Thus, infection can track longitudinally with little resistance, leading to rapid neurological deterioration.^1^ Consequently, management requires not only early identification via MRI with contrast, which is vital for delineating infection extent and neural compression, but also careful interpretation, as distinguishing subdural from epidural collections can be challenging even for experienced radiologists. Subtle or equivocal imaging findings should not dissuade clinicians from maintaining a high index of suspicion when the patient’s presentation suggests a rapidly progressive spinal infection. Early surgical decompression allows for effective drainage and debridement, while preserving neural integrity. Empiric broad-spectrum antibiotics should be initiated promptly and later refined based on culture results.

This case highlights the importance of emergency physicians maintaining a broad differential diagnosis in patients presenting with back pain, infectious symptoms, or neurologic deficits. Although rare, case reports and small series consistently emphasize spinal subdural abscesses’ high morbidity, mortality, and poor prognosis. Many patients develop permanent paraplegia or die.^12^, ^13^, ^14^, ^15^ Broader reviews similarly note disproportionately severe neurologic disability and mortality with subdural compared with epidural infections.^16^^,^^17^ Our patient’s T2 to L5 collection, with rapid progression from radiculopathy to multilevel neurologic dysfunction, illustrates the potential for extensive longitudinal spread and profound neurologic compromise even when early symptoms are nonspecific. Though the emergency physician may not distinguish spinal subdural abscesses from other spinal infections in the ED, awareness that spinal subdural abscesses may demonstrate more aggressive or widespread involvement may help contextualize rapidly progressive or pan-spinal disease. Additionally, this case serves as another reminder of the importance of timely identification of red flag signs and symptoms in patients presenting with back pain.

Back pain is a common complaint in the ED. Signs such as focal weakness or sensory deficits, bowel or bladder dysfunction, saddle anesthesia, unexplained weight loss, history of malignancy, recent trauma, anticoagulant or chronic steroid use, fever, immunosuppression, or IV drug use may indicate serious underlying pathology. These can generally be categorized as cauda equina syndrome, malignancy, vertebral fracture, infection, or abdominal aortic aneurysm. A focused neurologic examination, including assessment of motor strength, sensation, reflexes, straight-leg raise, rectal tone when indicated, and PVR, is critical. Notably, PVR is the most sensitive indicator for cauda equina syndrome, with a PVR <200 mL demonstrating a 97% negative predictive value.^18^

## 4 Conclusion

In conclusion, this case highlights the importance of early recognition of red flag signs and symptoms in patients with back pain and possible infection while detailing unique aspects of spinal subdural abscesses among spinal infections. Although spinal subdural abscess is rare, it highlights the potential for rapid progression and extensive neurologic involvement within this disease spectrum. Prompt, diffuse imaging of the entire spine and specialist consultation are critical in suspected spinal infections.^9^^,^^19^

## Ethics declaration

Written informed consent to take part in the study and to publish the article has been obtained from all participants or their legal representatives. The privacy rights of participants have been observed.

This study was performed in compliance with relevant laws, regulatory frameworks and guidelines where the research took place. Ethics committee approval was not required under relevant laws and institutional guidelines. Per Corewell Health research guidelines, single patient case reports do not require ethics committee review.

This research follows the CARE guidelines and the CARE checklist.

## Funding and Support

By *JACEP Open* policy, all authors are required to disclose any and all commercial, financial, and other relationships in any way related to the subject of this article as per ICMJE conflict of interest guidelines (see www.icmje.org). The authors have stated that no such relationships exist. This research did not receive any specific grant from funding agencies in the public, commercial, or not-for-profit sectors.

## Data Sharing Statement

This is a single-patient case report. De-identified clinical details and images sufficient to support the findings are included in the manuscript and figures. No additional datasets were generated or analyzed. Further de-identified information is available from the corresponding author on reasonable request with appropriate approvals and privacy safeguards.

## Conflict of Interest

All authors have affirmed they have no conflicts of interest to declare.
